# Stigma, Situational Triggers, and Symptoms: How Providers Justify Borderline Personality Disorder Among Sexual and Gender Minority Individuals

**DOI:** 10.1002/pmh.70012

**Published:** 2025-03-01

**Authors:** Anita Shubert, Najia Griffin, August Mashburn, Spirit Dorsey, Craig Rodriguez‐Seijas

**Affiliations:** ^1^ University of Michigan Ann Arbor Michigan USA

**Keywords:** borderline personality disorder, diagnostic bias, implicit bias, LGBTQ+ psychiatric disparities

## Abstract

Borderline personality disorder (BPD) is disproportionately diagnosed among sexual and gender minority (SGM) individuals relative to cisgender heterosexuals. However, research aimed at understanding the reasons for this disparity is scarce. The current study employed a mixed‐methods design to understand how mental healthcare providers' justifications for the BPD diagnosis differ based on the SGM status of the patient described and their own professional backgrounds. Two hundred seventy‐one providers who were randomly assigned to one of three identical vignette conditions, with SGM status manipulated, indicated their agreement with the BPD diagnosis and explained the reason for their agreement. Results from thematic content analyses illustrated that providers referenced three themes when explaining their agreement with the BPD diagnosis: (1) BPD as provisional, (2) BPD as certain, and (3) BPD criteria met. Providers referenced a greater variety of themes to explain their disagreement with the diagnosis: (1) situational factors, (2) insufficient time course, (3) diagnostic criteria unmet, (4) insufficient assessment information, (5) differential diagnosis, (6) developmental immaturity, and (7) stigma concerns. None of these justifications were differentially employed based on the SGM status of the vignette. However, differences were observed based on providers' backgrounds; psychologists more frequently cited concerns about time course, developmental immaturity, and having insufficient assessment information than psychiatrists, counselors, and social workers in disagreeing with the BPD diagnosis. Implications for reducing BPD diagnostic bias are discussed.

## 1 Introduction

Borderline personality disorder (BPD) is a psychiatric condition, the diagnosis of which is associated with myriad detrimental outcomes. Individuals diagnosed with BPD face stigma from both providers and the public; terms like “crazy,” “difficult,” “manipulative,” and “problematic” are often used to describe individuals with BPD (Aviram, Brodsky, and Stanley 2006; Masland et al. 2022). The population prevalence of BPD ranges from 1.2% in the general population to 12% in outpatient settings and 22% in inpatient settings (Ellison et al. 2018). Sexual and gender minority (SGM) individuals—those whose sexual orientations and gender identities do not conform with dominant heterosexual orientation or cisgender identities—experience a higher prevalence of the BPD diagnosis and symptomatology when compared with their cisgender heterosexual counterparts (Chang et al. 2021; Denning et al. 2022; Dulit et al. 1993; Paris, Zweig‐Frank, and Guzder 1995; Reich and Zanarini 2008; Rodriguez‐Seijas, Morgan, and Zimmerman 2021a; Singh, McMain, and Zucker 2011; Zimmerman, Benjamin, and Seijas‐Rodriguez 2022; Zubenko et al. 1987).

Some scholarship has suggested that the environmental stressors that SGM individuals experience could partially explain this elevated prevalence (Asadi, Suzuki, and Rodriguez‐Seijas 2025; Goldhammer, Crall, and Keuroghlian 2019; Rodriguez‐Seijas, Rogers, et al. 2023). Other scholarship has examined bias from mental healthcare providers representing a predilection to assign the BPD diagnosis when faced with an SGM individual as a presenting client (Assaad and Samuel 2022; Eubanks‐Carter and Goldfried 2006; Rodriguez‐Seijas, Morgan, and Zimmerman 2021b; Rodriguez‐Seijas, Warren, et al. 2024; Rodriguez‐Seijas, Morgan, and Zimmerman 2023). The goal of the current study pertains to this latter topic: to better understand the reasons that mental healthcare providers give to justify (dis)agreement with the BPD diagnosis and whether these justifications differ as a function of the SGM identity of the presenting client or the professional background of the respondent.

### 1.1 Bias in the Diagnosis of BPD Among SGM Individuals

Five studies, to date, have examined BPD diagnostic bias among SGM populations. Two studies demonstrated that providers assign the BPD diagnosis more frequently among SGM psychiatric patients, with disparities being unexplained by group‐related elevations in maladaptive personality domains thought to underlie the BPD diagnosis (Rodriguez‐Seijas, Morgan, and Zimmerman 2021b; Rodriguez‐Seijas, Morgan, and Zimmerman 2023). Other research using these same data demonstrate that—unlike the case for SGM individuals—discrepancies in BPD diagnosis based on race/ethnicity can be explained by group differences in these maladaptive personality domains (Becker et al. 2023).

Three studies have adopted a vignette‐based approach to examining SGM BPD diagnostic bias (Assaad and Samuel 2022; Eubanks‐Carter and Goldfried 2006; Rodriguez‐Seijas, Warren, et al. 2024). In these studies, identical patient vignettes are given to mental healthcare providers with SGM status being the only manipulated variable, from which providers may base their diagnostic decisions. Although Eubanks‐Carter and Goldfried (2006) found that clinicians more frequently diagnosed BPD among vignettes reflecting a gay/bisexual man, Assaad and Samuel (2022) found no significant differences in BPD diagnosis based on sexual minority status. Both studies of diagnostic bias, however, exclusively focused on cisgender vignettes. Rodriguez‐Seijas, Warren, et al. (2024) found bias in the diagnosis of BPD exclusively when providers were given a vignette depicting a transgender woman patient compared with vignettes depicting cisgender heterosexual or gay male patients. Further, this bias seemed most related to clinicians' perceptions of the individual's primary clinical diagnosis, and it differed based on the professional background of the provider (i.e., psychologist vs. psychiatrist vs. mental health counselor/social worker).

Germane to the current study, the decision to assign the BPD diagnosis was frequently misaligned with providers' decisions about whether the patient described met five or more BPD diagnostic criteria (i.e., diagnostic threshold). Regardless of SGM status or the professional background of participants, providers assigned the BPD diagnosis far less than they agreed that the patient met diagnostic threshold. Moreover, this *under*diagnosis of BPD differed based on the SGM status of the vignette and the professional background of participants. Psychologists were hesitant to assign BPD when presented with a transgender patient relative to their agreement that the described patient met diagnostic threshold. When presented with a patient who was cisgender—be it heterosexual or gay man—psychiatrists were hesitant to assign BPD even if they reported that the patient met threshold; they did not demonstrate this apparent hesitation when the patient was transgender. On the other hand, mental health counselors and social workers were hesitant to assign BPD even if they believed that diagnostic threshold was met only when the patient described was cisgender and heterosexual. So, although there seems to be a predilection to diagnose BPD based on the SGM status of the presenting individual, it appears particularly complex and worth further investigation.

### 1.2 Potential Biases in BPD Diagnostic Decision‐Making

Two biases that might be particularly relevant for understanding discrepancies in BPD diagnosis are the overpathologizing and confirmation biases (Bornstein and Emler 2001; Elstein 1988; López 1989; Snowden 2003). The overpathologizing bias refers to the interpretation of unfamiliar behaviors of marginalized group members as manifestations of psychopathology. Among SGM individuals, typical behavioral indicators of BPD criteria might operate differently when compared with cisgender heterosexuals. For instance, casual sexual behavior can be conceptualized as an indicator of the BPD impulsivity criterion. However, sexual behavior among SGM individuals might be motivated by such things as community belongingness (Jaffe et al. 2021) or the more normative use of dating apps as a means of accessing safe social spaces that might be otherwise difficult to access due to societal stigma (Chan 2023). Indeed, previous research demonstrates that endorsement of casual sexual behaviors as an indicator of the BPD diagnosis is less associated with psychopathology among sexual minority individuals relative to heterosexuals (Asadi, Suzuki, and Rodriguez‐Seijas 2025; Rodriguez‐Seijas, Rogers, et al. 2024). Further, sexual minority individuals more frequently endorse behaviors indicative of *all* BPD diagnostic criteria. However, they often endorse these behaviors independent of associated distress or impairment (Rodriguez‐Seijas, Morgan, and Zimmerman 2021a), the latter being necessary for the diagnosis. When provided with identical clinical descriptions, providers might be more inclined to view behaviors as reflective of the BPD diagnosis for SGM individuals than cisgender heterosexuals, reflecting the overpathologizing bias.

Confirmation bias occurs when evidence is gathered and interpreted in a way that confirms a diagnosis, rather than holistically considering evidence that could confirm or contradict the diagnosis. Historically, transgender identity has been equated with the BPD diagnosis within the psychiatric literature (Kavanaugh and Volkan 1978; Lothstein 1980, 1984; Meyer 1982; Murray 1985; Volkan and Berent 1976). Providers' justifications for a BPD diagnosis might serve to confirm previously held biases related to the historical equating of BPD with transgender and gender‐diverse identities. And this might occur as a function of the SGM status of the patient being described as well as the professional background of the respondent. Indeed, previous scholarship has speculated that the relative differences in training foci for psychologists compared with psychiatrists—and differences in ascription to the medical model—might impact differences in BPD diagnosis (Rodriguez‐Seijas, Warren, et al. 2024).

### 1.3 The Current Study

Previous research has examined differences in BPD diagnostic frequencies and bias among patients of different sexual orientations and gender identities. However, no literature has directly examined *why* mental healthcare providers do or do not agree with the BPD diagnosis in these cases. The current study, therefore, sought to answer the question: How do clinicians justify their BPD diagnostic decisions as a function of patient SGM status? To fill the gap in previous literature, we used a mixed methods approach to analyze qualitative data from a sample of mental healthcare providers who participated in a vignette‐based study of BPD diagnostic bias (Rodriguez‐Seijas, Warren, et al. 2024). The current study builds on previous research of provider bias in the diagnosis of BPD among SGM populations specifically by exploring (1) the various reasons that providers give for diagnosing/not diagnosing BPD and how these reasons differ based on (2) the SGM status of the patient described as well as (3) participants' professional backgrounds.

## 2 Method

### 2.1 Participants

Participants (*N* = 465) were practicing adult mental health professionals in the United States and Canada recruited between November 2021 and September 2022 as a part of a larger study on BPD diagnostic bias (Rodriguez‐Seijas, Warren, et al. 2024). Participants were recruited through professional listservs (e.g., Society for a Science of Clinical Psychology, Association for Behavioral and Cognitive Therapies, and National Association of Social Workers forum), medical schools, and regional professional organizations (e.g., US State Psychological Associations). Participants were invited to complete a screener indicating their current licensure status and were invited to participate once licensure information was verified.

Participants were excluded from data analysis if they failed two or more of the four attention check questions (*n* = 22) or if they only completed the demographic questions or took less than 5 min to complete the survey (*n* = 18). Finally, participants were excluded if they did not respond to the open‐ended explanation for their BPD diagnostic agreement (*n* = 154); the majority (*n* = 115) of those who did not respond to the open‐ended explanation for their BPD diagnostic agreement were clinical/counseling psychology graduate students in their 4th year of training or beyond. These students were not asked to provide open‐ended responses because they had not yet completed their terminal degree. The analytic sample, therefore, comprised *n* = 271 participants who provided qualitative information describing why they agreed or disagreed with the BPD diagnosis when presented with the case vignette, with a mean age of 39.02 years with a standard deviation of 12.42 years. Sample demographic information is presented in Table 1. All participants were compensated $10 for their involvement in the study and then entered a raffle to win one of six $90 gift cards. This study was approved by the University of Michigan Institutional Review Board.

**TABLE 1:** Demographic information of study participants.

	Cisgender heterosexual vignette		Sexual minority vignette		Gender minority vignette		Full sample	
	*n*	%	*n*	%	*n*	%	*n*	%
Gender identity								
Man	42	46.15	43	48.31	34	37.36	119	43.91
Woman	49	53.85	43	48.31	57	62.64	149	54.98
Non‐binary/gender fluid	0	0	1	1.12	0	0	1	0.37
Not listed	0	0	2	2.24	0	0	2	0.74
Race/ethnicity								
White	67	73.62	63	70.79	62	68.13	192	70.85
Black or African American	15	16.48	14	15.73	18	19.78	47	17.34
Hispanic	4	4.4	5	5.62	7	7.69	16	5.9
American Indian/Alaska Native	2	2.2	0	0	0	0	2	0.74
Asian	3	3.3	11	12.36	8	8.79	22	8.12
Middle Eastern/North African	1	1.1	1	1.12	1	1.1	3	1.11
Not listed	1	1.1	1	1.12	1	1.1	3	1.11
Current professional status								
Clinical/counseling psychologist	19	20.88	20	22.47	21	23.08	60	22.14
Psychiatrist	17	18.68	20	22.48	17	18.68	54	19.92
Mental health counselors/clinical social workers	55	60.44	49	55.05	53	58.24	157	57.93
Sexual orientation								
Heterosexual	73	80.22	71	79.78	80	87.91	238	87.82
Gay/lesbian	11	12.09	12	13.48	3	3.30	26	9.59
Bisexual	6	6.59	2	2.25	8	8.79	16	5.9
Not listed	1	1.1	4	4.49	0	0	5	1.85
Therapeutic orientation								
Psychodynamic	35	38.46	38	42.70	34	37.36	107	39.48
Feminist	10	10.99	8	8.99	16	17.58	34	12.55
Cognitive behavioral	65	71.43	63	70.79	71	78.02	233	85.98
Interpersonal	33	36.26	27	30.34	29	31.87	89	32.84
Not listed	17	18.68	11	12.36	12	13.19	40	14.76

*Note: N* = 271 (*n* = 91 for CH vignette, *n* = 89 for SM vignette, *n* = 91 for GM vignette). Participants were on average 39.02 years old (SD = 12.42), and participant age did not differ by vignette condition.

### 2.2 Measures

#### 2.2.1 Clinical Vignettes

Participants were randomly assigned to one of three clinical vignette conditions. The vignette described a fictional patient named Jesse who presented for an intake. The vignettes described experiences of identity concealment, social isolation, rejection sensitivity, heavy alcohol use, and suicidal thoughts and past behaviors, which align with common SGM experiences of psychosocial dysfunction (see Rodriguez‐Seijas, Warren, et al. 2024 for more details). The three vignettes were identical except for Jesse's gender identity and sexual orientation, resulting in the three vignette conditions: (1) cisgender heterosexual (CH) man (*n* = 91), (2) cisgender sexual minority (SM) man (*n* = 89), and (3) transgender heterosexual woman (*n* = 91) referred to as the gender minority (GM) vignette. The relationship between provider type and vignette condition was not significant, *χ* ^2^(4) = 0.76, *p* = 0.94.

#### 2.2.2 BPD Diagnosis Agreement

After reading the assigned vignette, participants were asked to indicate their level of agreement with the statement “Jesse should be diagnosed with borderline personality disorder” on a 4‐point Likert scale (strongly disagree, somewhat disagree, somewhat agree, strongly agree). We dichotomized this variable to reflect agreement (strongly agree, agree) and disagreement (strongly disagree, disagree) with the BPD diagnosis.

Among the clinical/counseling psychologists, 21 (35.00%) agreed with the BPD diagnosis and 39 (65.00%) disagreed with the BPD diagnosis. Among the psychiatrists/psychiatry residents, 34 (62.96%) agreed with the BPD diagnosis and 20 (37.04%) disagreed with the BPD diagnosis (see Table 2). Among the clinical social workers/mental health counselors, 111 (70.70%) agreed with the BPD diagnosis and 46 (29.30%) disagreed with the BPD diagnosis. Psychiatrists/psychiatry residents [*χ* ^2^ (1, *N* = 114) = 8.90, *p* = 0.003] and clinical social workers/mental health counselors [*χ* ^2^ (1, *N* = 217) = 23.22, *p* < 0.001] more frequently agreed with the BPD diagnosis than clinical/counseling psychologists.

**TABLE 2:** Participants' agreement with the BPD diagnosis as a function of vignette condition and professional status.

Provider type	Cisgender heterosexual vignette				Sexual minority vignette				Gender minority vignette			
	Agree		Disagree		Agree		Disagree		Agree		Disagree	
	*n*	%	*n*	%	*n*	%	*n*	%	*n*	*%*	*n*	%
Clinical/counseling psychologist	7	36.84	12	63.16	6	30.00	14	70.00	8	38.10	13	61.90
Psychiatrists/psychiatry residents	9	52.94	8	47.06	14	70.00	6	30.00	11	64.71	6	35.29
Clinical social workers/mental health counselors	34	61.82	21	38.18	34	69.39	15	69.39	43	81.13	10	18.87

#### 2.2.3 BPD Diagnosis Explanation

Participants were then asked to provide a written explanation for their BPD diagnostic agreement in an open‐ended format. The only instruction provided to respondents was “Please explain, in as much detail as you feel like sharing, why you [strongly agree, agree, disagree, strongly disagree] with the borderline personality disorder diagnosis for Jesse.”

#### 2.2.4 Analytic Strategy

The goals of the current study were to first use thematic content coding to understand the various justifications employed by participants for agreeing/disagreeing with the BPD diagnosis. Thereafter, we were interested in exploring if the employment of various justifications differed as a function of (1) the SGM status of the patient described in the vignette and (2) the professional background of participants. All quantitative statistical analyses were conducted using SPSS version 29.

##### 2.2.4.1 Thematic Coding Analysis

Three coders (AS, AM, SD) analyzed providers' written justifications of their BPD diagnostic agreement. Providers' demographic information, their assigned vignette condition, and their professional background were masked from coders during thematic analysis; the only information provided to coders was whether the written justifications were reflective of agreement or disagreement with the BPD diagnosis. We adopted a recursive process of thematic analysis consisting of six phases: familiarization with the data, coding, searching for themes, reviewing themes, defining and naming themes, and writing up the final codebook (Braun and Clarke 2006). First, members of the coding team reviewed all available data and developed an initial codebook. Then, all team members met to discuss and refine the initial themes. The three coders then used the refined coding scheme to determine reliability in use of the initial codebook. Following initial reliability testing, all team members met to discuss discrepancies in coding and to refine the codebook. This was done for two rounds, as reliability was below the acceptable benchmark of ≥ 70%. Following the finalization of the codebook, interrater reliability was calculated using a percent agreement calculation given the number of coders and subthemes identified. The bivariate percent agreement between the three coders ranged from 90.48% to 94.29% for participant responses in agreement with the BPD diagnosis and 95.78%–98.19% for participant responses that disagreed with the BPD diagnosis.

##### 2.2.4.2 Quantitative Analyses

After finalization of the codebook and the establishment of interrater reliability, we compared the frequencies of use for each identified theme as a function of vignette condition and participant professional background. To do so, we used chi‐square tests of independence to compare theme frequencies between individuals in the CH vignette condition with those in the SM and GM vignette conditions separately. We adopted this approach as previous findings from these data showed that BPD diagnostic bias was specific to the transgender and not the cisgender SM condition (Rodriguez‐Seijas, Warren, et al. 2024). Thus, collapsing the SM and GM conditions to a superordinate SGM group would not be appropriate. To examine the impact of participants' professional backgrounds, we compared theme frequencies among clinical/counseling psychologists with those from psychiatrists and mental health counselors/clinical social workers separately. Previous findings demonstrated that psychiatrists, mental health counselors, and clinical social workers were more likely to diagnose BPD across all conditions when compared with clinical/counseling psychologists (Rodriguez‐Seijas, Warren, et al. 2024).

## 3 Results

### 3.1 Thematic Analysis Findings

Descriptions and examples of themes based on participants' justifications of their agreement with the BPD diagnosis are presented in Table 3; justifications of disagreement with the BPD diagnosis are presented in Table 4.

**TABLE 3:** Participants' justifications for their agreement with the BPD diagnosis.

Theme	Description	Representative quote
Theme 1: Provisional diagnosis	Participants reflected that although they reported agreeing with the BPD diagnosis, they still experienced hesitancy about assigning it. They described being inclined to agree with the diagnosis but desiring additional information in order to be completely certain that BPD was appropriate.	“I do feel he has BPD, but I hesitate to dx someone with any PD, especially BPD, in just the first session. I would want more points of data over time to make such a dx.” [Psychologist, SM vignette]
Theme 2: Certainty of the BPD diagnosis	Participants reflected a level of certainty in their agreement with the BPD diagnosis.	“Jesse has alot of borderline personality disorder and i reallly support my assessment with the fact that jesse has been antisocial since childhood while being driven by success. Jesse further more cannot keep meaningful relationships and needs help to recover from emotional breakdowns.” [Counselor/Social Worker, SM vignette]
Theme 3: BPD diagnostic criteria met	Participants commented on the specific BPD diagnostic criteria that were met in justifying their agreement with the BPD diagnosis.	“Based on my read of the vignette, Jesse appears to satisfy the criteria (in no particular order) of identity disturbance, impulsivity, recurrent suicidality, and unstable/intense interpersonal relationships.” [Psychologist, SM vignette]

**TABLE 4:** Participants' justifications for their disagreement with the BPD diagnosis.

Theme	Description	Representative quote
Theme 1: Situational factors better explain presenting problems	Participant responses reflected a belief that the situational factors experienced by the patient (Jesse) were better explanations of the presenting psychopathology, thereby making the BPD diagnosis inappropriate.	“This assessment occurs in the context of a significant transition in life for Jesse during which time they are determine who they are. This in, in turn, naturally shapes their interactions with others and relationships with others. In this context, components of this may be developmentally appropriate given their current stage in life and the transition they are going through.” [Psychiatrist, CH vignette]
Theme 2: Insufficient information regarding time course	Participants reflected a belief that a lack of sufficient information documenting the temporal stability of the symptoms being described rendered the BPD diagnosis inappropriate.	“I disagree with the diagnosis not due to a lack of qualifying symptomology (with subjective interpretation), but strictly due to the timeframe in which it's being provided. Conceptually, BPD is possible given the client's reported history, however, it would not be conclusive until further evaluation. I'd provide a provisional diagnosis until assessments can be administered, such as the MSI‐BPD, and until a pattern of behavior can be clarified and established.” [Psychiatrist, SM vignette]
Theme 3: BPD diagnostic criteria unmet	Participants reflected a belief that a lack of sufficient information documenting the temporal stability of the symptoms being described rendered the BPD diagnosis inappropriate.	“The vignette describes some features that might be consistent with borderline personality disorder but there is not enough information available to make a clear cut diagnosis and the available information could also occur with other diagnoses. Of the information that is available, the magnitude of symptoms and severity of psychopathology seem less than typically seen with borderline personality disorder. The number and pattern of borderline features also seems less prominent than I would expect for a borderline PD diagnosis. Thus, borderline traits may be present but I do not think they rise to the threshold of an actual diagnosis. Also, because of the stigma associated with a borderline personality disorder diagnosis, my tendency is to be cautious about jumping to conclusions about borderline PD based only on a small amount of information, particularly when someone is in crisis.” [Psychiatrist, CH vignette]
Theme 4: Insufficient assessment information	Participants reflected that there was insufficient information for them to feel comfortable providing a BPD diagnosis.	“Not enough information to diagnose BPD. Need much more historical data and current data regrading pt's current level of fxning and baseline level of fxning (to whatever degree such information is possible to ascertain); additional data needed to assign diagnoses of BPD would, at a minimum, include information reported by patient during session, clinician observation of pt during, btwn, and across sessions, assessment data (ideally outcome monitoring data collected/tracked over time), eported by/collected from pt during sessions, reported by patient in session, assessment data/outcomes monitoring data (tracked over time), and collaboration/input from other providers or reliable historians/narrators (if possible).” [Psychologist, GM vignette]
Theme 5: Differential diagnosis	Participants identified a different psychiatric disorder diagnosis as more appropriate than the BPD diagnosis.	“I think adjustment disorder is more appropriate as he had. A recent event that worsened potentially preexisting mood dysphoria. He does not present with splitting behaviors” [Counselor/Social Worker, CH vignette]
Theme 6: Developmental maturation incomplete	Participants' justifications reflected a belief that the patient's developmental maturation processes were incomplete, attributing presenting dysfunction to this immaturity rather than a BPD diagnosis.	“It's still early on in his dating and psychosexual developmental history. He's basically just starting to come out to himself and those most important to him (his family) so this pattern of short and unstable relationships are probably acute and time‐limited due to his mood disorder. Its too soon to diagnose a personality disorder. Would need more time and data to support that diagnosis.” [Psychologist, SM vignette]
Theme 7: Stigma concerns	Participants highlighted concerns about the stigma affiliated with the BPD diagnosis in justifying their disagreement with the appropriateness of the diagnosis.	“They show some signs of it, but I would be very cautious in assigning anyone a personality disorder. There are a lot of intense, negative connations with having one, and I would never diagnosis it in a first session without being absolutely certain.” [Counselor/Social Worker, GM vignette]

### 3.2 BPD Diagnostic Agreement Themes

We identified three themes illustrating how providers justified their agreement with the BPD diagnosis: (1) BPD as a provisional diagnosis, (2) certainty of the BPD diagnosis, and (3) belief that the vignette demonstrated criteria for BPD diagnosis (see Table 3).

#### 3.2.1 BPD as a Provisional Diagnosis

Participants mentioned that they agreed with the BPD diagnosis. However, they expressed hesitancy about assigning it after only one interaction with a patient.

I do feel he has BPD, but I hesitate to dx someone with any PD, especially BPD, in just the first session. I would want more points of data over time to make such a dx. [Psychologist, SM vignette]

Similarly, several participants indicated hesitancy about the BPD diagnosis due to the stigma associated with the diagnosis, demonstrating awareness of the implications of a BPD diagnosis and the importance of being certain of its presence.

BPD is a highly stigmatized diagnosis, and treatment recommendations for BPD (Dialectical Behavioral Therapy) is an intensive program. I would want more than one session with Jesse prior to making a BPD diagnosis, looking out for additional information on the severity and pervasiveness of his symptoms and for information that might highlight rule‐out diagnoses (e.g., dependent personality) or concerns that warrant intervention ahead of his interpersonal difficulties (e.g., suicidality, alcohol use). [Psychologist, CH vignette]

Some respondents also indicated hesitancy about assigning the BPD diagnosis due to concerns about how situational factors affected the clinical presentation.

Jesse appears to meet diagnostic criteria for BPD, although I would not conceptualize this as their primary dx. It would be very important to consider how Jesse's upbringing and experience with Gender dysphoria also contribute to some of their BPD sxs (e.g., identity development, relationship instability, experience with their emotions/emotional regulation skills). [Psychologist, GM vignette]

#### 3.2.2 Certainty of the BPD Diagnosis

This theme reflected participants' expressed certainty in the appropriateness of the BPD diagnosis.

He matches the patterns of behavior consistent with this diagnosis, both in terms of meeting technical diagnostic criteria and overall clinical picture. Symptoms are consistent with diverse impairment in multiple domains that is also suggestive of broad‐based underlying diagnosis. [Psychiatrist, SM vignette]

#### 3.2.3 BPD Diagnostic Criteria Met

This theme illustrated some participants' tendencies to highlight the BPD diagnostic criteria that they believe were met in justifying their agreement with the BPD diagnosis. Participants often listed five or more BPD diagnostic criteria, demonstrating consideration of the diagnostic threshold necessary for the BPD diagnosis.

Affective instability, series of impulsive short lasting relationships, emotional lability, sense of emptiness, impulsive, suicidal gestures. [Psychiatrist, CH vignette]

Participants sometimes also referenced the personality disorder diagnostic criterion of temporal stability in justifying their agreement with the BPD diagnosis.

The fact that at least some of his reported symptoms have been consistently causing him difficulty dating back to high school also weighs into my thinking that these are chronic symptoms that manifest in several different important areas of life. [Psychologist, SM vignete]

### 3.3 BPD Diagnostic Disagreement Themes

We identified seven themes in participants' justifications for their disagreement with the BPD diagnosis: (1) Situational factors better explain presenting problems, (2) insufficient information regarding time course, (3) BPD diagnostic criteria unmet, (4) insufficient assessment information, (5) differential diagnosis, (6) developmental maturation incomplete, and (7) stigma concerns (see Table 4).

#### 3.3.1 Situational Factors Better Explain Presenting Problems

This theme reflected participants' beliefs that external contextual factors like family life, religion, and SGM minority stress processes explained the presenting concerns. Their justifications implied that the stressful environmental contexts in which Jesse finds himself/themself rendered a BPD diagnosis inappropriate.

I think that Jesse's experiences are pretty understandably grounded in the context of his experiences. He is closeted to his family because of (understandable) fears that they will not approve of him. [Psychologist, SM vignette]

Relationship distress and emerging sense of self are not necessarily pathological in a transitional age youth who is also navigating differences between family of origin values and his own values as he forges his own path. [Psychiatrist, CH vignette]

#### 3.3.2 Insufficient Information Regarding Time Course

Participants disagreed with the BPD diagnosis on the bases of not meeting the time course of BPD due to the lack of previous history, insufficient length of symptom presentation, or expressing doubt that the current symptoms will persist into the future. These participants disagreed that the symptoms described met the personality disorder criterion of temporal stability.

I think that Jesse does not have the chronic pattern of BPD symptoms at this point. [Psychologist, SM vignette]

It is not clear that the cluster B traits described in the prompt have persisted over the course of a lifetime or that his unstable relationship patterns are evident outside of sexual relationships. [Psychiatrist, CH vignette]

#### 3.3.3 BPD Diagnostic Criteria Unmet

This theme represented participants' beliefs that insufficient BPD diagnostic criteria were met to justify the diagnosis.

… First, there is only instance of suicidal gesture, need at least 2 to be recurrent. Moreover it could be explained moreso by depression than BPD; Anger criterion does not meet full threshold for me, his anger/irritability is reasonable and only one outburst was described. Impulsivity is also within reason and reactive to the breakups, though potentially this is subthreshold (and the drinking meets more of the crtierion than the sexual impulsivity); The relationships piece does not say "abandonment" issues to me … Identity disturbance seemed subthreshold to me, but warrants further assessment here. No transient stress‐related symptoms. Didn't get much of an "emptiness" vibe from the vignette … [Psychologist, CH vignette]

#### 3.3.4 Insufficient Assessment Information

In this theme, respondents stated that there was insufficient information provided by the vignette, and thus, there was a need for further assessment to confidently diagnose BPD. In contrast with those who were inclined to agree with BPD as a provisional diagnosis—warranting further assessment—this theme illustrated a tendency to disagree, rather than agree, with the BPD diagnosis without this additional information.

I do not know Jesse well enough to diagnosis a personality disorder and do not feel enough information is given … I would only diagnosis BPD with further assessment and after knowing Jesse longer … [Counselor/Social Worker, SM vignette]

There was not enough information from that vignette for me to diagnose a personality disorder. Also, I usually base those diagnoses based of my clinical interactions with the patient (i.e. how their personality shape the therapeutic relationship). [Psychologist, CH vignette]

#### 3.3.5 Differential Diagnosis

Some respondents expressed the belief that a different diagnosis was more appropriate than the BPD diagnosis. Respondents often identified such disorders as social anxiety disorder, major depressive disorder, generalized anxiety disorder, or a substance use disorder as more appropriate than the BPD diagnosis.

I see Jesse as more depressed and anxious with a potential alcohol use disorder. [Psychologist, GM vignette]

I think adjustment disorder is more appropriate as he had. A recent event that worsened potentially preexisting mood dysphoria. He doesn't present with splitting behaviors. [Counselor/Social Worker, CH vignette]

#### 3.3.6 Developmental Maturation Incomplete

This theme represents participants' beliefs that something about the developmental process was incomplete. This immature development—such as the patient's age, the patient's ongoing identity development, or the patient's brain immaturity—is cited as rendering the BPD diagnosis inappropriate; these internal personal processes better explained the behaviors than the BPD diagnosis.

Jesse is early in identity development and is approrpiately experiencing issues related to dating and intimacy with others. [Counselor/Social Worker, SM vignette]

They are experiencing many recent changes and while they have had some unstable relationships they are fairly young, their identity is still developing, and they are transitioning from a sheltered environment where emotions and being yourself was viewed as not okay. Much of what many experience in adolescence appears to be what Jesse is now experiencing … [Psychologist, GM vignette]

Although similar, this theme differs from the insufficient information regarding time course. References to incomplete development maturation were about the patient's identity development and age, rather than the chronicity of BPD symptoms, which is represented in the insufficient time course theme.

#### 3.3.7 Stigma Concerns

This theme reflected participants' hesitancy to assign the BPD diagnosis due to stigma associated with the diagnosis and concerns about the long‐term implications of diagnosing BPD for the patient.

… BPD is a controversial diagnosis, as many have noted that some who are diagnosed with BPD might better be understood as coping with trauma … It can be a stigmatizing diagnosis and I would also not want it to live in his medical chart forever if I have any hesitation about the diagnosis. [Counselor/Social Worker, SM vignette]

… . Also, because of the stigma associated with a borderline personality disorder diagnosis, my tendency is to be cautious about jumping to conclusions about borderline PD based only on a small amount of information, particularly when someone is in crisis. [Psychiatrist, CH vignette]

### 3.4 Quantitative Comparisons of Thematic Frequencies

Frequencies of each theme based on vignette condition and professional background are presented in Table 5.

**TABLE 5:** Frequencies of thematic references in participants' justifications, stratified by vignette condition and participant professional background.

Theme	Frequencies											
	Cisgender heterosexual vignette		Sexual minority vignette		Gender minority vignette		Psychologist		Psychiatrist		Counselor/social worker	
	*n*	%	*n*	%	*n*	%	*n*	%	*n*	%	*n*	%
Agreement with the BPD diagnosis												
Provisional diagnosis	20	40.00	13	24.07	17	27.42	12	57.14	17	50.00	21	18.92
Certainty of the BPD diagnosis	3	6.00	6	11.11	4	6.45	1	4.76	2	5.88	10	9.01
BPD diagnostic criteria met	40	80.00	44	81.48	50	80.65	16	76.19	26	76.47	92	82.88
Disagreement with the BPD diagnosis												
Situational factors	13	31.70	20	57.14	10	34.48	16	41.03	7	35.00	20	43.48
Insufficient time course	8	19.51	6	17.14	4	13.79	11	28.21	3	15.00	4	8.70
BPD diagnostic criteria unmet	15	36.59	7	20.00	7	24.14	13	33.33	7	35.00	9	19.57
Insufficient assessment information	15	36.59	13	37.14	11	37.93	21	53.85	5	25.00	13	28.26
Differential diagnosis	12	29.27	9	25.71	7	24.14	8	20.51	3	15.00	17	36.96
Developmental maturation incomplete	8	19.51	7	20.00	7	24.14	10	25.64	8	40.00	4	8.70
Stigma concerns	2	4.88	6	17.14	5	17.24	4	10.26	1	5.00	8	17.39

*Note:* Frequencies of thematic references do not equal the number of participants or responses as individual participant responses might contain more than one theme.

#### 3.4.1 Vignette SGM Status

There were no significant differences in the frequencies in which various themes were used to justify either agreement or disagreement with the BPD diagnosis based on whether the vignette reflected a sexual or gender minority individual compared with the cisgender heterosexual vignette condition.

#### 3.4.2 Participant Professional Background

##### 3.4.2.1 Agreement With the BPD Diagnosis

Clinical/counseling psychologists' justifications for their agreement with the BPD diagnosis more frequently reflected the theme of the diagnosis being provisional when compared with mental health counselors' and clinical social workers', *χ* ^2^ (1, *N =* 132) = 13.76, *p* < 0.001.

##### 3.4.2.2 Disagreement With the BPD Diagnosis

Psychologists more frequently referenced the themes of insufficient information about time course, *χ* ^2^ (1, *N* = 85) = 5.53, *p* = 0.019, and incomplete developmental maturation, *χ* ^2^ (1, *N* = 85) = 4.41, *p* = 0.036, when compared with mental health counselors and clinical social workers. Psychologists also more frequently referenced the theme of having insufficient assessment information than psychiatrists, *χ* ^2^ (1, *N* = 59) = 4.46, *p* = 0.035, and mental health counselors and clinical social workers, *χ* ^2^ (1, *N* = 85) = 5.76, *p* = 0.016, respectively.

## 4 Discussion

This mixed methods study examined how mental healthcare providers justify their agreement with the BPD diagnosis and whether the relative employment of these justifications differed based on the SGM identity of the patient described and the professional background of the providers. Results from our thematic analysis demonstrated that in considering the appropriateness of the BPD diagnosis—even in cases where providers were inclined to agree that BPD was warranted—providers were often concerned about not only fidelity to the diagnosis in terms of major criteria but also temporal stability, developmental maturity, and the breadth of the assessment. Additionally, providers frequently cited concerns about the stigma that could result from the BPD diagnosis and how social context could impact presenting psychopathology. However, we did not find evidence of SGM bias in terms of differential use of justifications. We did, however, find that psychologists were more inclined to cite concerns about time course, developmental maturity, and a perception of insufficient information more frequently than other professions in explaining their disagreement with the BPD diagnosis. These results provide further understanding of the nature of BPD diagnostic bias, specifically in relation to SGM populations. They further underscore the importance of fidelity in the training of psychiatric assessment and diagnosis.

Our previous findings demonstrated that when patients presented with identical clinical profiles, transgender women and patients seen by psychiatrists, mental health counselors, and clinical social workers—regardless of sexual orientation or gender identity—were more likely to be given a BPD diagnosis or to view BPD as the principal presenting concern (Rodriguez‐Seijas, Warren, et al. 2024). Based on this, we hypothesized that specific biases might be at play—and thus evidenced in the justifications that mental healthcare providers describe—when deciding on the appropriateness of the BPD diagnosis. And we surmised that these biases might be differentially enlisted based on the SGM status of the patient being described. However, providers in this study did not demonstrate provider bias. That is, there were no differences across vignette conditions in providers' justifying agreement with the BPD diagnosis based on meeting diagnostic threshold differentially as a result of the SGM status. Neither did we observe any evidence that would be congruent with confirmation bias (i.e., implying that the gender minority vignette was more representative of BPD consistent with historical equating of gender minority identity and the BPD phenotype). Thus, although providers appear more inclined to agree with the BPD diagnosis when presented with a vignette representing a transgender client, relative to vignettes reflecting cisgender heterosexual or gay clients, there were no specific justifications that explained this pattern. It would seem that this bias to diagnose BPD preferentially among transgender clients—all other things like symptom presentation being equal—might be largely implicit.

Our results suggest that some signal exists in relative differences in justifications for disagreeing with the BPD diagnosis based on mental healthcare providers' professional backgrounds. However, these findings are still incongruent with our previous speculations on this topic. For instance, we suggested that one reason for differences in diagnostic patterns among psychologists, psychiatrists, mental health counselors, and clinical social workers would reflect relatively different areas of training emphasis (Rodriguez‐Seijas, Warren, et al. 2024). For instance, we previously compared the American Psychological Association's (2012, 2015) relatively exhaustive guidelines for working with SGM individuals outlining how stigma and other contextual factors compromise SGM individuals' mental health with the comparatively briefer guidelines from the American Psychiatric Association (*Working with LGBTQ Patients*, n.d.), in which less attention is devoted to environmental considerations. Based on this speculation, we would expect that psychologists would employ justifications reflecting situational factors or concerns about stigma preferentially when compared with psychiatrists. However, there were no differences across professional groupings on the employment of these justifications in explaining disagreement with the BPD diagnosis. Instead, our results highlight that psychologists were more inclined to reference concerns about time course, developmental maturity, and a perception of insufficient information more frequently than other professions in explaining their disagreement with the BPD diagnosis. It is unclear if the hesitance to agree with the BPD diagnosis among psychologists, relative to other mental health professionals, is specific to the BPD diagnosis or more generalized.

These results add to previous findings that diagnostic bias appears to arise at the psychiatric disorder diagnosis level rather than being reflected in the assignments of diagnostic criteria (Morey and Benson 2016; Morey and Ochoa 1989; Rodriguez‐Seijas, Warren, et al. 2024). What our findings add to the literature is that there seems to be—at least among our diverse sample of mental healthcare providers—no specific discernable rationale that explains SGM diagnostic bias in relation to the BPD diagnosis. Thus, the best intervention that we can suggest at this time in efforts to counteract potential bias in the diagnosis of BPD among SGM populations is a reminder that providers ensure that they base the BPD diagnosis explicitly on the diagnostic criteria that are met rather than perceptions that any clinical presentation represents an essentialized narrative of the BPD phenotype. Providers should ensure that they can explicitly account for each diagnostic criterion they consider present. Additionally, that SGM individuals often exhibit behaviors considered indicators of all nine BPD diagnostic criteria, though without the necessary distress/impairment (Asadi, Suzuki, and Rodriguez‐Seijas 2025), it is important to reiterate that providers ensure that the behaviors are impairing, and not simply differences that might be attributed to group‐specific normative processes. Beyond the BPD criterion A diagnostic symptoms, providers are reminded to also ensure that the criterion of temporal stability is sufficiently met and to rule out that presenting symptoms are not better explained by an individual's culture (p. 629), behavioral responses to stress (p. 630), or environmental factors (p. 763).

### 4.1 Limitations

This study is not without its limitations. First, our vignette was not designed as a definitive description of the BPD phenotype. Instead, we adopted the approach consistent with Eubanks‐Carter and Goldfried (2006) illustrating several clinical domains that could be expected among SGM individuals presenting for psychiatric treatment and that also map onto theoretical connections between SGM minority stress processes as well as the BPD diagnostic domains. Second, we only assessed three vignette conditions, all of which reflected individuals assigned male at birth. Third, how providers respond based on a clinical vignette might differ from diagnostic decisions made in the real world. Fourth, our sample was relatively homogenous with approximately 70% of the sample in each vignette condition reporting white race. Finally, we did not assess participants' experiences working with individuals with a BPD diagnosis, which might impact diagnostic decision‐making in this type of study.

## 5 Conclusion

The current study used a mixed‐methods approach to identify themes that underlie mental healthcare providers' agreement with the BPD diagnosis. We also investigated how the use of these justifications differed as a function of the SGM status of the patient being described as well as the professional backgrounds of the healthcare providers. Our results revealed that providers employed myriad justifications for their diagnostic decisions. However, we found no evidence that they preferentially used any specific justifications based on the SGM status of the patient. Providers, however, differed in their use of these justifications as a function of their professional backgrounds. Relative to psychiatrists, mental health counselors, and clinical social workers, clinical and counseling psychologists more frequently referenced themes about having insufficient information to make the BPD diagnosis and concerns that the patient described was developmentally immature to justify their disagreements with the BPD diagnosis.

## Conflicts of Interest

The authors declare no conflicts of interest.

## Data Availability

The data that support the findings of this study are available from the corresponding author upon reasonable request.
